# Cybersicherheit in Krankenhäusern – Teil 1: IT-Compliance als Leitungsaufgabe

**DOI:** 10.1365/s43439-022-00049-8

**Published:** 2022-04-13

**Authors:** Diana Nadeborn, Tilmann Dittrich

**Affiliations:** 1 Tsambikakis & Partner Rechtsanwälte mbB, Schlüterstr. 39, 10629 Berlin, Deutschland; 2 Heinrich-Heine-Universität, Düsseldorf, Deutschland

**Keywords:** Cyberangriff, Gesundheitsversorgung, Kritische Infrastrukturen, Lösegeldzahlung, Cyberversicherung, Cyber attacks, Healthcare, Critical infrastructure, Ransom payments, Cyber insurance

## Abstract

Die Gefahr von Cyberangriffen ist aktuell in sämtlichen Branchen allgegenwärtig. Bei Krankenhäusern kann ein Cybervorfall neben dem Verlust von Patientendaten sogar zu einer Gefahr für Leib und Leben der Patienten führen. Krankenhäuser sind daher durch eine Vielzahl von Gesetzen zur Vorhaltung ausreichender Schutzmaßnahmen verpflichtet. Die Kontrolle der Einhaltung dieser Vorschriften obliegt den Leitungspersonen in Krankenhäusern. Es handelt sich hierbei um eine wichtige Compliance-Aufgabe. Ein Baustein kann in diesem Zusammenhang der Abschluss einer Cyberversicherung sein. Besondere Vorsicht ist bei der Zahlung von Lösegeldern im Fall einer Ransomware-Attacke geboten.

## Abstract (en)

The threat of cyber attacks is a burning issue in all industries. In the case of hospitals, a cyber incident can not only lead to the loss of patient data but can also put patients’ lives in danger. Hospitals are therefore obliged by a large number of laws to maintain sufficient protective measures. Monitoring compliance with these regulations is the responsibility of hospital managers. This is an important compliance task. Cyber insurance can be part of the solution. Particular caution is required when paying ransoms in the event of ransomware attacks.

## Einleitung

Lücken in der IT-Sicherheit erleichtern Kriminellen Angriffe auf das IT-System eines Krankenhauses. „Cyberkriminelle greifen dort an, wo es sich aus ihrer Sicht finanziell lohnt. Besonders wirtschaftlich starke Unternehmen, Kritische Infrastrukturen (KRITIS) und öffentliche Einrichtungen (z. B. Krankenhäuser), die unter dem Begriff ‚Big Game‘ zusammengefasst werden, sind hoch gefährdet.“^1^

Hacker nutzen ihren Zugriff auf die Daten, um Krankenhäuser zu erpressen. Zunächst verschlüsseln sie die Daten des Unternehmens und verlangen die Zahlung eines Lösegelds für die Herausgabe der Entschlüsselungscodes. Solange die Daten verschlüsselt sind, können Patienten nicht umfassend medizinisch versorgt werden. Außerdem kopieren die Hacker die Daten und verlangen weitere Zahlungen, damit die Daten der Patienten nicht veröffentlicht bzw. weitergegeben werden. Cyberangriffe können damit nicht nur zu einem Ausfall der IT-Systeme führen, sondern auch das Patientenwohl und die Patientensicherheit gefährden.

Krankenhäuser müssen deshalb für IT-Sicherheit sorgen und sich vor Cyberangriffen schützen. Da Cyberangriffe mit enormen wirtschaftlichen Schäden und Gefahren für die Reputation des Unternehmens einhergehen können, ist die Geschäftsleitung dafür zuständig, durch geeignete Maßnahmen die Cybersicherheit des Unternehmens aufzubauen und zu erhalten.

Nachfolgend soll zunächst die als hoch einzustufende Cyberbedrohungslage speziell für Krankenhäuser und der daraus resultierende Handlungsbedarf dargestellt werden. Anschließend soll der Umfang der IT-Compliance-Pflicht für Leitungspersonen in Krankenhäusern, die durch eine Vielzahl an Vorschriften reguliert ist, im Einzelnen erläutert werden. In diesem Zusammenhang wird auch auf Compliance-Risiken bei Lösegeldzahlungen und einer etwaigen Compliance-Pflicht zum Abschluss einer Cyberversicherung eingegangen. In einem Anschlussbeitrag „Cybersicherheit in Krankenhäusern – Teil 2: Vom Normalfall zum Notfall“ werden die konkret notwendigen Maßnahmen und Pflichten der Krankenhäuser im Zusammenhang mit der Cybersicherheit nach BSIG und DS-GVO behandelt.

## Handlungsbedarf für Krankenhäuser

Im Dezember 2021 bestimmte eine Warnmeldung des Bundesamtes für Sicherheit in der Informationstechnik (BSI) über mehrere Tage das Mediengeschehen: Es hatte Kenntnis über die Softwareschwachstelle „Log4Shell“ erlangt und diesbezüglich die Warnstufe Rot ausgerufen.^2^ Wenige Tage später erfolgten bereits die ersten Meldungen über Cybervorfälle in Unternehmen, die auf diese Schwachstelle zurückgeführt wurden.^3^ So ist es üblich, dass Cyberkriminelle nach Bekanntwerden solcher Schwachstellen versuchen, diese für Angriffe und Erpressungen auf Unternehmen und Privathaushalte auszunutzen. Von solchen Cyberbedrohungen sind mittlerweile sämtliche Branchen betroffen. Im Gegensatz zu den meisten Branchen kommt es aber im Gesundheitswesen nicht „nur“ zu wirtschaftlichen Schäden durch die Störung des Betriebsablaufs bei Cybervorfällen, sondern auch zu Gefährdungen von Patienten. Wegen dieser Bedrohungslage sehen Experten die Umsetzung von IT-Sicherheitsmaßnahmen im Gesundheitssektor als ungenügend an.

### Viele Krankenhäuser verwundbar

Im Frühjahr 2021 wurde auf der Cycon-Konferenz der NATO ein Forschungsbericht von drei Wissenschaftlern (vom IT-Dienstleister *Alpha Strike Labs*, von der *Universität der Bundeswehr *und vom IT-Dienstleister *Limes Security*) über die Angriffsfläche („attack surface“) deutscher Krankenhäuser während der Covid-19-Pandemie im Jahr 2020 vorgestellt [1]. Die Forscher hatten die Cybersicherheitslage von etwa 1500 deutschen Krankenhäusern mittels einer Vulnerabilitäts-Systemanalyse untersucht. Sie kamen zum Ergebnis, dass knapp ein Drittel der untersuchten Systeme verwundbar sind [1, S. 83 und S. 87]. Bemerkenswert ist, dass bei Krankenhäusern mit mehr als 30.000 vollstationären Fallzahlen pro Jahr, also Kritischen Infrastrukturen nach § 6 BSI-KritisV, mehr Schwachstellen in den IT-Systemen festgestellt werden konnten als bei kleineren Krankenhäusern. Das dürfte auch darauf zurückzuführen sein, dass dort mehr vernetzte IT-Systeme genutzt werden [1, S. 89 und S. 92]. Die Autoren der Studie stellten bereits bei der IT-Ausstattung große Probleme fest. So werden teilweise IT-Systeme verwendet, für die kein Support mehr vom Hersteller bereitgestellt wird. Einer der Autoren gibt zu bedenken, dass es am Risikobewusstsein inklusive ausreichender Budgetierung innerhalb der Gesundheitseinrichtungen für eine ausreichende Cyberresilienz fehlen könnte. Weiterhin müsse überdacht werden, ob der Staat nicht bei der Schwachstellensuche selbst aktiv werden sollte.^4^ Denn das systematische Ausnutzen dieser Schwachstellen könne zu einem nationalen Sicherheitsrisiko in Deutschland führen [1, S. 92 f.]. So sind die Auswirkungen eines flächendeckenden Ausfalls von Krankenhäusern in einer Region offenkundig, wenn nicht einmal andere Krankenhäuser als Redundanzen für ein angegriffenes Krankenhaus dienen können.

### Keine ausreichenden IT-Sicherheitsmaßnahmen

Der IT-Dienstleister *Kaspersky* führte im Jahr 2021 durch *Airlington Research *eine Umfrage unter 150 Führungskräften in Deutschland und jeweils 100 Führungspersonen in Österreich und der Schweiz im Gesundheitswesen mit dem Titel „Patient Krankenhaus. Kaspersky-Studie zur IT-Sicherheitslage im Gesundheitswesen in Deutschland, Österreich und der Schweiz“ durch.^5^ Etwa 60 % der Befragten sind in Unternehmen mit 50 bis 1000 Mitarbeitern tätig, die weiteren Befragten in Unternehmen mit größeren Mitarbeiterzahlen. Knapp Dreiviertel der befragten Krankenhäuser in Deutschland ist während der Covid-19-Pandemie bereits Opfer eines Cyberangriffs geworden. Etwa zwei Drittel der befragten Führungskräfte aus Deutschland bezeichnete die getroffenen Cybersicherheitsmaßnahmen als ausreichend. Gleiches galt für die Frage, ob Unternehmen für Krisenfälle ein ausreichendes Notfallkonzept (Business Continuity Plan) vorhalten würden. Etwa ein Drittel der befragten Teilnehmer aus dem deutschen Gesundheitswesen gab an, dass die Belegschaft ihres Unternehmens über ausreichende IT-Sicherheitskenntnisse verfügen würde. Abschließend beurteilten die Befragten, dass von ihren Mitarbeitern die größte Gefahr für Cybervorfälle ausgehen würde. Immerhin ein Drittel der Befragten hat in der eigenen Wahrnehmung keine ausreichenden Sicherheitsmaßnahmen getroffen und ist nicht auf Krisenfälle vorbereitet. Nicht zu Unrecht werden Mitarbeiter als Hauptrisiko ausgemacht. Dennoch werden IT-Sicherheitsschulungen vernachlässigt.^6^

### Gefährdung der Patientensicherheit

Große Aufmerksamkeit erregte ein Cybervorfall am Uniklinikum Düsseldorf im Jahr 2020, der im Lagebericht des BSI zur Cybersicherheit in Deutschland als warnendes Praxisbeispiel für die Gefahren von Cyberangriffen angeführt wurde.^7^ Das Großklinikum musste sich nach einem Hackerangriff für mehrere Wochen von der Notfallversorgung abmelden. Eine Patientin verstarb im Zusammenhang mit dem Cyberangriff, da sie in Düsseldorf nicht aufgenommen werden konnte und ein anderes Zielklinikum angefahren werden musste.^8^ Nachdem das Klinikum publik gemacht hatte, dass es Opfer des Angriffs geworden war, gaben die Täter die Entschlüsselungsmöglichkeiten ohne Lösegeldzahlung heraus. Die zuständigen Ermittlungsbehörden gingen daher davon aus, dass die Täter das Klinikum nur versehentlich getroffen haben und der Angriff eigentlich der Heinrich-Heine-Universität Düsseldorf gelten sollte.

### Erhöhung der Verwundbarkeit durch Vernetzung

Auch wenn Krankenhäuser nicht direktes Ziel eines Angriffs sind, können sie indirekt von einem Cybervorfall betroffen sein.^9^ So muss das IT-Sicherheitsmanagement eines Krankenhauses auch darauf ausgerichtet sein, dass Produkte oder externe Dienste, die das Krankenhaus im Rahmen der Behandlung nutzt, ausfallen können. Laut der erwähnten Kaspersky-Umfrage sahen nur rund 7 % Gefahren durch externe Partner in der Lieferkette.^10^ Im Zusammenhang mit der „Log4Shell“-Schwachstelle musste die gematik GmbH im Dezember 2021 Teile der Telematikinfrastruktur (TI), also eines Raums, der in Zukunft den Datenaustausch zwischen Leistungserbringern im Gesundheitswesen ermöglichen soll, außer Dienst nehmen.^11^ In Zukunft, wenn die TI flächendeckend etabliert ist, könnte ein solcher Vorfall dazu führen, dass Krankenhäuser plötzlich die Anwendungen der TI nicht mehr nutzen können, also bspw. keine Verordnungen mehr ausstellen und nicht mehr mit anderen Leistungserbringern über *Kommunikation im Medizinwesen* (KIM) kommunizieren können. Es bedarf dann eines Auffangkonzepts für die Versorgungsschritte während der Behandlung.

## IT-Compliance-Pflicht für Leitungspersonen in Krankenhäusern

Unter Compliance versteht man die Pflicht von Leitungspersonen in Unternehmen, sich selbst rechtskonform zu verhalten und zu verhindern, dass aus dem Unternehmen heraus, also von Mitarbeitenden, Rechtsverstöße begangen werden. Hergeleitet wird die Pflicht zur Compliance aus der allgemeinen Sorgfalt der Leitungspersonen, § 43 GmbHG, §§ 93, 76 AktG.

Kommt es aufgrund von Compliance-Verstößen zu Schäden beim Unternehmen oder Dritten, drohen den Leitungspersonen Haftungsansprüche. Hinzu kommen Aufsichtspflichten aus dem OWiG, die sowohl für die Leitungspersonen selbst als auch für Unternehmen zu empfindlichen Geldbußen führen können, §§ 130, 30 OWiG [3, § 2 Rn. 10 ff.]. Dieses aktuell noch unter dem Opportunitätsprinzip stehende Sanktionsregime könnte in Zukunft durch ein Unternehmensstrafrecht/Verbandssanktionengesetz mit Geltung des Legalitätsprinzips abgelöst werden, wovon der Gesetzgeber sich wirksamere Sanktionen von Unternehmensrechtsverletzungen erhofft.^12^ Dadurch sollen regionale Unterschiede bei der Anwendung des OWiG in Deutschland behoben, höhere Bußgeldsanktionen möglich und die Berücksichtigung von Compliance-Maßnahmen durch die Unternehmen deutlicher geregelt werden [6, S. 35 ff.].

Für die konkrete Ausgestaltung der Compliance-Pflicht und das zu ihrer Umsetzung etablierte Compliance-Management-System (CMS) ist es von besonderer Bedeutung, welche rechtlichen Risiken dem Unternehmen drohen, welche Auswirkungen von einem Rechtsverstoß ausgehen und wie wahrscheinlich der Risikoeintritt zu bewerten ist. Anhand einer regelmäßig durchgeführten Risikoanalyse werden die für die Unternehmenstätigkeit relevanten Rechtsvorschriften und ihre Beachtung im Unternehmen festgestellt.^13^

Der Cybersicherheit kommt im Rahmen dieser Risikoanalyse in einem Krankenhaus eine besondere Bedeutung zu. Sie ist durch eine Vielzahl an Vorschriften reguliert, aus denen sich erhebliche negative Rechtsfolgen ergeben können. Darüber hinaus ist die allgemeine Bedrohungslage, wie eingangs vorgestellt, aktuell als *sehr hoch* einzuschätzen.

Außerdem müssen Leitungspersonen berücksichtigen, dass sie zwar Compliance-Pflichten innerhalb des Unternehmens auf spezielle Abteilungen delegieren können. So kann die Einhaltung der besonderen Cybersicherheitsvorschriften etwa auf die IT-Abteilung delegiert werden. Eine Letztverantwortlichkeit verbleibt dennoch bei den Führungspersonen. Es bedarf demnach einer hinreichenden finanziellen Ausstattung und einer regelmäßigen Kontrolle der besonderen Abteilung. Da der Bereich der IT sich mit einer möglichen Compliance-Abteilung überschneidet, muss der Delegierende weiterhin darauf achten, dass die Aufgaben zwischen den Abteilungen klar verteilt und, wo notwendig, eine „Verzahnung“ der Zuständigkeiten vorgenommen wird.

### Spezielle Cybersicherheitsvorschriften

#### Organisatorische und technische Vorkehrungen nach § 8a BSIG

Ende 2020 hat die EU-Kommission ihre *EU Cybersecurity Strategy* veröffentlicht, mit der das Cybersicherheitsniveau in einem vernetzten Europa auf ein ausreichendes Schutzniveau gestellt werden soll. Teil der *EU Cybersecurity Strategy* ist der Entwurf einer NIS-2-RL, welche die bisherige Europäische Richtlinie zur Gewährleistung einer hohen Netzwerk- und Informationssicherheit (NIS-RL) zukünftig ablösen soll. Nach deren Verabschiedung soll auch das BSIG angepasst werden, welches von Krankenhäusern, die zur Kritischen Infrastruktur zählen, zu berücksichtigen ist.

Bei der Kategorie der *Kritischen Infrastrukturen* handelt es sich nach § 2 Abs. 10 S. 1 BSIG um Einrichtungen, die (unter anderem) dem Sektor Gesundheit angehören und für das Funktionieren des Gemeinwesens von hoher Bedeutung sind, da durch ihren Ausfall oder ihre Beeinträchtigung Gefährdungen für die öffentliche Sicherheit eintreten würden. Es geht also nicht nur um die alleinige Sicherheit digitaler Vorgänge, sondern vielmehr um den Schutz der Gesundheitsversorgung und Patientensicherheit. Zur Kritischen Infrastruktur gehören gem. § 6 BSI-KritisV Krankenhäuser mit mehr als 30.000 vollstationären Behandlungen pro Jahr. Hierzu gehören nur zugelassene Krankenhäuser i. S. d. § 108 SGB V. Zur Berechnung des Schwellenwerts kann auf die Daten nach § 21 Abs. 2 KHEntgG zurückgegriffen werden. Ist ein Krankenhaus auf mehrere Betriebsstätten verteilt, entscheidet der Landeskrankenhausplan, ob sie als ein Krankenhaus i. S. d. BSI-KritisV anzusehen sind.^14^

Nach § 8a Abs. 1 S. 1 BSIG sind die Betreiber Kritischer Infrastrukturen verpflichtet, angemessene organisatorische und technische Vorkehrungen (OTV) zur Vermeidung von Störungen der Verfügbarkeit, Integrität, Authentizität und Vertraulichkeit ihrer informationstechnischen Systeme, Komponenten oder Prozesse zu treffen, die für die Funktionsfähigkeit der von ihnen betriebenen Kritischen Infrastrukturen maßgeblich sind. Hierbei gilt es, den aktuellen Stand der Technik zu beachten.^15^

Neu hinzugekommen ist die Regelung des § 8a Abs. 1a BSIG, wonach die OTV ab dem 01.05.2023 auch den Einsatz von Systemen zur Angriffserkennung umfassen müssen. Die geeigneten Parameter und Merkmale aus dem laufenden Betrieb müssen kontinuierlich und automatisch erfasst und ausgewertet werden. Dadurch sollen die Kritischen Infrastrukturen in der Lage sein, fortwährend Bedrohungen zu identifizieren und zu vermeiden sowie für eingetretene Störungen geeignete Beseitigungsmaßnahmen vorzusehen. Nach § 2 Abs. 9b S. 2 BSIG soll die Angriffserkennung durch einen Abgleich der in einem informationstechnischen System verarbeiteten Daten mit Informationen und technischen Mustern erfolgen, die auf Angriffe hindeuten. Damit Unternehmen stets auf aktuelle Erkennungsmuster zurückgreifen können, hält das BSI eine *Malware Information Sharing Platform (MISP) *iRd § 8b Abs. 2 BSIG bereit.^16^

Eine bedeutende Rolle kommt nun den *branchenspezifischen Sicherheitsstandards (B3S)* zu. Hierbei handelt es sich um einheitliche Standards, die Betreiber Kritischer Infrastrukturen und ihre Branchenverbände zur Gewährleistung der OTV vorschlagen können und die anschließend durch das BSI als geeignet festgestellt werden. Dies hat den Vorteil, dass die unbestimmten Rechtsbegriffe der OTV konkretisiert werden. Kritikwürdig erscheint, dass im Gesetz nicht geklärt ist, wie lange die Feststellung des BSI über die Geeignetheit des B3S wirkt. So müssen die OTV der KRITIS-Betreiber dem Stand der Technik entsprechen, während nicht gesichert ist, dass auch der einschlägige B3S dem aktuellen Stand entspricht. Vernünftigerweise erfolgt laut BSI die Feststellung der Geeignetheit in der Regel nur für einen Zeitraum von zwei Jahren.^17^ Aktuell sollten die B3S durch die KRITIS-Betreiber auf ihre Aktualität geprüft und etwaige Abweichungen begründet und dokumentiert werden. Weiterhin sind die Branchenverbände angehalten, aktualisierte B3S beim BSI einzureichen und als geeignet feststellen zu lassen. Letztlich liegt es aber am Gesetzgeber, hier für Rechtssicherheit zu sorgen und ein „Ablaufdatum“ der B3S vorzuschreiben.

Für den Bereich der stationären medizinischen Versorgung ist Ende 2019 ein von der Deutschen Krankenhausgesellschaft (DKG) eingereichter B3S vom BSI als geeignet festgestellt worden.^18^ Der B3S baut auf gängigen IT-Sicherheits-Management-Systemen (u. a. DIN ISO 27001) auf und berücksichtigt die Voraussetzung der *Angemessenheit* der OTV dadurch, dass er „Muss‑, Soll- und Kann“-Anforderungen an die Krankenhäuser enthält.

Im Zusammenhang mit den OTV bestehen für die Betreiber der Kritischen Infrastrukturen Bußgeldrisiken nach § 14 BSIG, etwa wenn die OTV nicht, nicht richtig, nicht vollständig oder nicht rechtzeitig getroffen werden. Der Bußgeldrahmen kann nach § 14 Abs. 5 BSIG im Millionenbereich liegen mit der Möglichkeit der Verzehnfachung für Unternehmen nach § 30 Abs. 2 S. 3 OWiG. Dem Bußgeldkonzept steht durch die geplante NIS-2-RL eine Annäherung an das Sanktionsregime der DS-GVO mit der Bemessung am Jahresumsatz eines Konzerns bevor [8, S. 214 (217)]. Es muss von den Unternehmen beobachtet werden, ob sich hieraus entgegen der bislang zurückhaltenden Bußgeldpraxis des BSI eine Steigerung der Compliance-Risiken ergibt [9].

#### Organisatorische und technische Vorkehrungen nach § 75c SGB V

Nicht nur größere Krankenhäuser, die zur Kritischen Infrastruktur gehören, müssen für IT-Sicherheit sorgen. Durch das PDSG^19^ wurde mittlerweile für alle Krankenhäuser eine ausdrückliche Verpflichtung zum Ergreifen von IT-Sicherheits-Maßnahmen in das SGB V aufgenommen. Der Wortlaut in § 75c Abs. 1 SGB V zu den OTV, die Krankenhäuser ab dem 01.01.2022 treffen müssen, ist nahezu deckungsgleich zu § 8a BSIG.^20^ Für die Angemessenheit der OTV können sich die Krankenhäuser nach § 75c Abs. 2 SGB V an den im Rahmen des § 8a Abs. 2 BSIG als geeignet festgestellten B3S orientieren. Ein solches Vorgehen unter Berücksichtigung der oben genannten Schwächen des B3S ist dringend zu empfehlen. Die Verpflichtung gilt nach § 75c Abs. 3 SGB V für alle Krankenhäuser, soweit sie nicht ohnehin als Betreiber Kritischer Infrastrukturen gemäß § 8a BSIG angemessene technische Vorkehrungen zu treffen haben. Der Verzicht auf den Begriff der „organisatorischen“ Vorkehrungen dürfte ein Redaktionsversehen sein. § 75c SGB V konkretisiert die allgemeinen Compliance-Pflichten von Leitungspersonen, sodass sich aus einer Verletzung Haftungsansprüche ergeben können.

#### Technische und organisatorische Maßnahmen nach Art. 32 DS-GVO

Der Datenschutz bildet einen Teil der Cybersicherheit ab. Nach Art. 5 Abs. 1 lit. f DS-GVO muss bei der Datenverarbeitung unter den Stichworten *Integrität und Vertraulichkeit* eine angemessene Sicherheit für personenbezogene Daten vor einer unbefugten bzw. unrechtmäßigen Verarbeitung sowie vor unbeabsichtigtem Verlust, unbeabsichtigter Zerstörung oder unbeabsichtigter Schädigung durch geeignete technische und organisatorische Maßnahmen (TOM) gewährleistet werden. Bereits durch den Wortlaut wird klar, dass sich hieraus Vorgaben für die Cybersicherheit von Krankenhäusern und anderen Leistungserbringern ergeben. Art. 32 DS-GVO konkretisiert die allgemeine Verpflichtung von Art. 5 Abs. 1 DS-GVO zur Sicherheit bei der Datenverarbeitung und den Anforderungen an die TOM. So gibt Art. 32 Abs. 1 DS-GVO Anwendungsbeispiele für TOM vor. Aufgrund der Sensibilität von Gesundheitsdaten sind die aufgeführten TOM von Krankenhäusern zwingend zu beachten. So spielt die Verschlüsselung von Daten eine wichtige Rolle im Gesundheitswesen, aber auch die dauerhafte Funktionsfähigkeit und Resilienz mit Notfallplänen (Business Continuity Management bzw. IT-Service Continuity Management) sowie die regelmäßige Überprüfung und Evaluation der getroffenen TOM. Die oben aufgeführte Bedrohungslage macht aber deutlich, dass vor allem auch der organisatorische Teil der Sicherheitsmaßnahmen nicht zu unterschätzen ist. So muss ein klar vorgegebenes Aufgaben- und Beauftragungssystem zur Umsetzung der datenschutzrechtlichen Vorgaben im Unternehmen mit regelmäßigen Schulungen, hinreichender finanzieller Ausstattung und etabliertem Berichtswesen aufgrund der Compliance-Delegation vorgehalten werden.

Die datenschutzrechtlichen Aspekte der Cybersicherheit stellen für die Unternehmen im Gesundheitswesen aus mehreren Gesichtspunkten einen wichtigen Compliance-Bereich dar. Dies liegt zunächst an den unbestimmten Rechtsbegriffen im Zusammenhang mit den TOM. Trotz der Konkretisierung durch Art. 32 Abs. 1 DS-GVO verbleibt ein Interpretationsspielraum, der zu einer Rechtsunsicherheit hinsichtlich der tatsächlichen Umsetzung von TOM führt. Zudem fehlt es bislang an hilfreicher Rechtsprechung zu den TOM. Zu beachten sind daher vor allem die Publikationen und Empfehlungen der Landesdatenschutzbehörden im Zusammenhang mit den TOM im Gesundheitswesen.^21^

Hinzu kommen die beträchtlichen Folgen eines Datenschutzverstoßes, die sowohl die Justiz als auch die Literatur in letzter Zeit umfassend beschäftigt haben. So ist etwa im Rahmen der Schadensersatzansprüche nach Art. 82 DS-GVO die Beweis- und Darlegungslast der einzelnen Anspruchsvoraussetzungen und Konkretisierung des immateriellen Schadensersatzes umstritten, während die Datenschutzbehörden bereits die ersten Bußgelder nach Art. 83 DS-GVO in Millionenhöhe ausgesprochen haben.^22^ Es wird wohl einer Entscheidung des EuGH bedürfen, wie konkret die Behörden in den Unternehmen Aufsichtspflichtverletzungen von Leitungspersonen bei Datenschutzverstößen nachweisen müssen, um Bußgelder gegen die Unternehmen aussprechen zu können.^23^ Im Vergleich zu den Rechtsfolgen des BSIG lässt sich im Rahmen von Art. 5, 32 DS-GVO feststellen, dass hier bereits eine Sanktionspraxis der zuständigen Behörden etabliert ist, weshalb das Risiko hinsichtlich der Einhaltung der DS-GVO aktuell als höher bewertet werden kann.

### Allgemeine Regelungen mit Berührung zu Cybervorfällen

Wenig Aufmerksamkeit in den Veröffentlichungen zur Cybersicherheit in Krankenhäusern wurde bislang der sogenannten *Krankenhausalarm- und -einsatzplanung (KAEP)* gezollt. Alle Krankenhäuser in Deutschland sind aufgrund von landesrechtlichen Vorschriften (Landeskrankenhausgesetze, zudem i. d. R. noch über Regelungen in den Rettungsdienst- und Katastrophenschutzgesetzen) zur Vorhaltung einer KAEP verpflichtet.^24^ So müssen Krankenhäuser für Großschadenslagen, die sich auch aus Cybervorfällen ergeben können, Alarmpläne vorhalten, die hinreichend eingeübt sind. Je nach Ausgestaltung müssen KAEP-Team und die IT-Abteilung daher vernetzt arbeiten. Hilfreich für die Einrichtung und Vorhaltung der KAEP sind die Empfehlungen des „Handbuchs KAEP“ des Bundesamts für Bevölkerungsschutz und Katastrophenschutz (BBK).^25^

Weiterhin unterliegen medizinische Leistungserbringer berufs- und strafrechtlichen Verschwiegenheitsverpflichtungen, die in § 9 MBO‑Ä bzw. in den jeweiligen Landesberufsordnungen und in § 203 StGB verankert sind. Im Zuge dieser Regelungen sind Berufsgeheimnisträger auch dazu verpflichtet, hinreichende Sicherheitsvorkehrungen zu treffen, damit Dritte nicht unbefugt an Patientengeheimnisse gelangen können. Unterlassen sie diese Sicherheitsmaßnahmen, kann hierin der Vorwurf eines berufsrechtswidrigen bzw. strafbaren Unterlassens liegen.^26^

Wie im Fall des Düsseldorfer Uniklinikums beschrieben, besteht immer auch das Risiko von Patientenschädigungen aufgrund von Cybervorfällen. So können vernetzte Systeme in Krankenhäusern ausfallen, an die der Patient unmittelbar angeschlossen ist. Dazu gehört beispielsweise der Einsatz von Telemonitoring, das der Überwachung von Patienten aus der Distanz dient. Möglich sind auch unvorhergesehene Patientenverlegungen aufgrund von Cybervorfällen, die sich schädlich auf das Patientenwohl auswirken können. In Düsseldorf musste die Notfallversorgung abgemeldet und dadurch Patienten in weiter entfernte Krankenhäuser transportiert werden. Kommt es aufgrund solcher Verzögerungen zu Patientenschädigungen, werden straf- und zivilrechtliche Konsequenzen relevant. Im Bereich der Körperverletzungs- oder auch Tötungsdelikte spielt die Cybersicherheit bei der Bemessung eines Fahrlässigkeitsmaßstabs eine Rolle. So leitete die zuständige Staatsanwaltschaft im Fall des Düsseldorfer Uniklinikums Ermittlungen gegen Unbekannt wegen fahrlässiger Tötung nach § 222 StGB ein. Es ließ sich jedoch nicht nachweisen, dass die Patientin aufgrund des längeren Fahrweges verstorben war, sodass das Verfahren mittlerweile wieder eingestellt wurde.^27^ Es ist aber auch nicht ausgeschlossen, dass sich solche Ermittlungen gegen Verantwortliche von Krankenhäusern richten, wenn der Verdacht unzureichender IT-Sicherheit aufkommt. Bei Schadensersatzansprüchen kommt erschwerend hinzu, dass es sich bei der Cybersicherheit um einen voll beherrschbaren Risikobereich i. S. d. § 630h Abs. 1 BGB handeln könnte und damit Beweiserleichterungen für die Patientenseite eintreten.

### Compliance-Risiken bei Lösegeldzahlungen aufgrund von Ransomware

Kriminelle verbinden Cyberattacken vielfach mit Lösegeldforderungen. Es ist auch möglich, dass sie mehrere solcher Forderungen an die betroffenen Unternehmen stellen. Häufig verlangen sie zunächst eine Geldzahlung zur Herausgabe von Verschlüsselungscodes. Oft können dann unter der Androhung der Veröffentlichung erbeuteter Daten, was im Gesundheitswesen aufgrund deren Sensibilität besonders dramatisch ist, weitere Lösegeldforderungen folgen, was aber auch unmittelbar angedroht werden kann. Für die Leitungspersonen stellt sich hier die Frage, ob sie solchen Forderungen nachkommen sollen. Denn damit könnte die Entschlüsselung der Daten erfolgen und die Betriebs- und Patientengefährdung beendet und eine mögliche Veröffentlichung sensibler Daten (mit damit einhergehenden Rechtsrisiken) vermieden werden. Allerdings setzt eine solche Lösegeldzahlung zunächst voraus, dass die von den Tätern typischerweise geforderte Kryptowährung vom Krankenhaus überhaupt zur Verfügung gestellt werden kann.^28^ Weiterhin ist auch nicht sicher, ob die Täter gewillt sind, auf eine Zahlung mit der angekündigten Preisgabe von Entschlüsselungscodes zu reagieren. Teilweise ist die Schadsoftware überhaupt nicht darauf ausgelegt. Zudem wäre die Annahme falsch, man werde aufgrund von Lösegeldzahlungen in Zukunft von Cyberangriffen einer Gruppierung verschont. Nachvollziehbarerweise wird vom Bundeskriminalamt abgeraten, solchen Lösegeldzahlungen Folge zu leisten, um kriminelles Handeln nicht noch zu belohnen.

Im Zusammenhang mit Lösegeldzahlungen werden immer wieder Strafbarkeitsrisiken diskutiert, die mit der Zahlung einhergehen sollen. Im Raum stehen dann etwa die Unterstützung einer kriminellen Vereinigung gem. §§ 129 Abs. 1 S. 2 StGB oder Verstöße gegen das Außenwirtschaftsgesetz gem. § 18 Abs. 1 AWG, wobei vor allem bei der Gefährdung von Menschenleben im Gesundheitsbereich eine Rechtfertigung gem. § 34 StGB in Betracht kommen soll [14, S. 575, 15, S. 281 (285 f.)]. Regelmäßig soll es auch schon am Vorsatz von Seiten des Lösegeld Zahlenden fehlen [16, S. 103 (106)]. Dabei mag es sich jedoch um einen eher akademischen Streit handeln. In der Praxis wird eine Ermittlungsbehörde, die aufgrund der Meldung des Betroffenen Kenntnis von einem Ransomware-Angriff erlangt, ihre Ermittlungsbemühungen auf die Identifizierung der zunächst unbekannten Täter konzentrieren. Parallel dazu geführte Ermittlungen gegen das geschädigte Unternehmen und dessen Leitungspersonen, welche die tatnächsten Zeugen in dem Verfahren gegen Unbekannt sind, sind bisher in keinem Erpressungsfall bekannt geworden.

### Schadensminderung durch Cyberversicherungen

Im Rahmen der Risikobewertung spielt es auch eine Rolle, welcher Versicherungsschutz im Unternehmen zur Absicherung von Compliance-Vorfällen besteht. Für Cybervorfälle kommt hierfür neben der bekannten „D&O“-Versicherung, die eine Haftpflichtversicherung zwischen Versicherer und Unternehmen zugunsten der Leitungspersonen darstellt, eine Cyberversicherung in Betracht [17, S. 247 (250)]. Laut einer Studie des Branchenverbands Bitkom e. V. aus dem Jahr 2018 verfügten rund ein Drittel aller Unternehmen in Deutschland mit mehr als 500 Mitarbeitern über eine Cyberversicherung. Etwa 30 % der Unternehmen mit Cyberversicherung, die in einem Zeitraum von zwei Jahren einen Cyberangriff erlitten hatten, gaben an, die Police habe sich ausgezahlt.^29^

Leider besteht für die Inhalte solcher Cyberversicherungen ein wahrer „Dschungel“ an Varianten bei den Versicherern.^30^ Vor Abschluss müssen die Versicherungsbedingungen mit den etwaigen Leistungsausschlüssen konsequent geprüft werden. Cyberversicherungen setzen sich regelmäßig aus verschiedenen Versicherungsschwerpunkten zusammen, weshalb sie auch als „multidisziplinäres Produkt“ mit Schwerpunkten aus der Haftpflichtversicherung, der Eigenschadendeckung, der Betriebsunterbrechung und einer Rechtsschutzversicherung bezeichnet werden.^31^

Gemäß der AVB Cyber^32^ deckt die Cyberversicherung Vermögensschäden, die durch eine Informationssicherheitsverletzung verursacht worden sind (*AVB Cyber A1‑1*) [20, S. 421 (422)]. Dies ist eine Beeinträchtigung der Verfügbarkeit, Integrität und Vertraulichkeit (sog. CIA-Triade) von elektronischen Daten des Versicherungsnehmers oder von informationsverarbeitenden Systemen, die er zur Ausübung seiner betrieblichen Tätigkeit nutzt (*AVB Cyber A1‑2*). Als Auslöser für diese Informationssicherheitsverletzung kommen Angriffe, unberechtigte Zugriffe oder Schadprogramme in Betracht (*AVB Cyber A1‑2.4*). Zu den Vermögensschäden zählen keine Personenschäden, was für den Gesundheitsbereich ein bedeutender Ausschluss ist und ggf. individuelle Vereinbarungen mit dem Versicherer notwendig macht (*AVB Cyber A1‑3*).

Vor Abschluss einer Cyberversicherung sind zudem deren Obliegenheiten und Leistungsausschlüsse zu beachten. So stellen die AVB Cyber eigenständige Anforderungen an die informationsverarbeitenden Systeme des Unternehmens auf (*AVB Cyber A1-16*). Hierzu gehören Schutzmaßnahmen durch Berechtigungskonzepte, erhöhte Schutzmaßnahmen bei Geräten, die über das Internet erreichbar sind, ein aktualisierter Schutz gegen Schadsoftware und engmaschige Backup-Verfahren (*AVB Cyber A1-16.1*). Zusätzlich besteht die Obliegenheit für den Versicherungsnehmer, alle gesetzlichen und behördlichen Sicherheitsvorschriften einzuhalten (*AVB Cyber A1-16.2*). Dies betrifft u. a. die bereits aufgezeigten Regelungen aus dem BSIG, dem SGB V und der DS-GVO. Weiterhin besteht die Obliegenheit, besonders gefahrdrohende Umstände auf Verlangen des Versicherers innerhalb angemessener Frist zu beseitigen. Rechtsfolge nicht eingehaltener Obliegenheiten ist eine Leistungsbefreiung bei vorsätzlicher Verletzung oder die Kürzung bei grob fahrlässiger Verletzung um die Schwere des Verschuldens (*AVB Cyber B3‑4.2*). Zusätzlich gelten allgemeine Leistungsausschlüsse. Diese betreffen vorvertragliche Informationssicherheitsverletzungen (*AVB Cyber A1-17.1*), Lösegeldzahlungen (*AVB Cyber A1-17.7*) und die durch vorsätzliches oder wissentliches Abweichen von Vorschriften verursachten Schäden (*AVB Cyber A1-17.10*).

Cyberversicherungen stellen also nur für solche Unternehmen ein taugliches Compliance-Element dar, die bereits umfassende Informationssicherheitsmaßnahmen entsprechend den geltenden Vorschriften ergriffen haben und professionell aufgestellt sind. Für andere Unternehmen dürfte die Möglichkeit einer Cyberversicherung auch praktisch daran scheitern, dass die Versicherer sich im Rahmen des § 19 VVG einen Eindruck über den Stand der Cybersicherheit im Unternehmen verschaffen und bei stetig steigenden Deckungssummen Abstand nehmen vom Abschluss einer Versicherung.^33^

## Fazit

In einem modernen Krankenhaus werden administrative Daten und Patientendaten elektronisch verwaltet, Spezialanwendungen für Labor, Radiologie oder Intensivstation eingesetzt und alle Anwendungssysteme miteinander vernetzt. Was eine Verbesserung in der Verwaltung und Versorgung der Patienten darstellt, ist zugleich Einfallstor für Cyberkriminelle, die in den Worten des Bundeskriminalamtes eine Großwildjagd („Big Game Hunting“) betreiben. Eine erfolgreiche und nachhaltige Digitalisierung setzt Informations‑ und Cybersicherheit voraus. Krankenhäuser sind aufgrund ihrer gesellschaftlichen Relevanz Adressaten besonders vieler gesetzlicher Pflichten. Dabei dient die IT-Compliance dem Schutz vor Hackerangriffen, der Patientensicherheit und schließlich auch dazu, das Risiko der Haftung und Bestrafung des Unternehmens beziehungsweise seiner Organe und Mitarbeiter zu reduzieren.

## Footnotes

1 *BKA*, Cybercrime Bundeslagebild 2020 (Stand: April 2021), S. 29 (abrufbar unter: https://www.bka.de/DE/AktuelleInformationen/StatistikenLagebilder/Lagebilder/Cybercrime/cybercrime_node.html; zuletzt abgerufen am: 11.01.2022).

2 https://www.bsi.bund.de/DE/Service-Navi/Presse/Pressemitteilungen/Presse2021/211211_log4Shell_WarnstufeRot.html (zuletzt abgerufen am: 07.01.2022).

3 https://www.handelsblatt.com/technik/it-internet/java-bibliothek-log4j-gefaehrliche-sicherheitsluecke-die-angriffe-auf-unternehmen-und-behoerden-beginnen-/27900826.html (zuletzt abgerufen am: 11.01.2022).

4 https://e-health-com.de/details-unternehmensnews/it-sicherheitsrisiko-krankenhaus/ (zuletzt abgerufen am: 26.12.2021).

5 https://go.kaspersky.com/healthcare-report.html (zuletzt abgerufen am 07.01.2022). Eine Übersicht zu den Studienergebnissen findet sich auf den S. 5 ff. des Abschlussberichts.

6 *Kaspersky*-Studie, S. 10.

7 *BSI*, Die Lage der IT-Sicherheit in Deutschland 2021, S. 15 (abrufbar unter: https://www.bsi.bund.de/DE/Service-Navi/Publikationen/Lagebericht/lagebericht_node.html; zuletzt abgerufen am 07.01.2022).

8 https://www.wired.co.uk/article/ransomware-hospital-death-germany (zuletzt abgerufen am: 11.01.2022).

9 Weitere Beispiele zur Gefahr aufgrund von Vernetzung vgl. [2, S. 100 ff.].

10 *Kaspersky*-Studie, S. 9

11 https://www.healthcare-computing.de/gematik-muss-teile-der-ti-vom-netz-nehmen-a-1083370/ (zuletzt abgerufen am: 21.12.2021).

12 Zum Entwurf eines „Verbandssanktionengesetzes“ aus dem Jahr 2020 vgl. u. a. [4, S. 205 (210), 5, S. 377].

13 *Theusinger/Jung*, in: MAH GmbHR, 4. Aufl. 2018, § 24 Rn. 38 ff [7].

14 *Jorzig/Sarangi*, Digitalisierung im Gesundheitswesen, 2020, S. 85 f.

15 Zu den weiteren kooperativen Anforderungen an die KRITIS-Betreiber wie Nachweis- und Meldepflichten im Zusammenhang mit den OTV siehe *Nadeborn/Dittrich*, Cybersicherheit in Krankenhäusern – Teil 2: Vom Normalfall zum Notfall, ICLR 2022, issue 2.

16 BT-Drs. 19/26106, S. 79.

17 https://www.bsi.bund.de/DE/Themen/KRITIS-und-regulierte-Unternehmen/Kritische-Infrastrukturen/Allgemeine-Infos-zu-KRITIS/Stand-der-Technik-umsetzen/Uebersicht-der-B3S/uebersicht-der-b3s_node.html (zuletzt abgerufen am: 19.01.2022).

18 https://www.dkgev.de/themen/digitalisierung-daten/informationssicherheit-und-technischer-datenschutz/informationssicherheit-im-krankenhaus/ (zuletzt abgerufen am: 02.01.2022).

19 Gesetz zum Schutz elektronischer Patientendaten in der Telematikinfrastruktur (Patientendaten-Schutz-Gesetz – PDSG) vom 14.10.2020 (BGBl. I, 2115).

20 Lediglich das Schutzziel der Authentizität ist nicht ausdrücklich genannt, was aber aufgrund der Öffnung für weitere Schutzziele und den B3S keine Auswirkungen haben dürfte.

21 Vgl. bspw. die „Best-Practice-Prüfkriterien zur Cybersicherheit für medizinische Einrichtungen“ des BayLFD (Stand: 27.05.2020) (abrufbar unter: https://www.datenschutz-bayern.de/technik/best_practices/; zuletzt abgerufen am: 11.01.2022).

22 Eine Übersicht zu den Schadensersatz-Entscheidungen gibt [10, S. 18]; eine Übersicht zu den bisherigen Bußgeldern gibt [11, S. 289].

23 KG, Beschl. v. 06.12.2021 – 3 Ws 250/21, BeckRS 2021, 39748.

24 Eine Übersicht zu den Rechtsgrundlagen der KAEP ist im „Handbuch KAEP“ des BBK, S. 22 f. abgebildet (Fn. 30), zur KAEP vgl. [12, S. 618–621].

25 https://www.bbk.bund.de/DE/Themen/Gesundheitlicher-Bevoelkerungsschutz/Krankenhausalarmplanung/krankenhausalarmplanung_node.html (zuletzt abgerufen am: 31.12.2021).

26 *Hassemer*, in: [13, § 43 Rn. 173]; *Schuster*, medstra 2015, 280 (281).

27 https://www.t-online.de/region/duesseldorf/news/id_88934068/duesseldorf-todesfall-nach-hackerangriff-an-klinik-ermittlungen-eingestellt.html (zuletzt abgerufen am: 11.01.2022).

28 vgl. BGH NJW 2021, 2301 (2302): Lösegeldforderungen über geldwerte PIN-Codes elektronischer Zahlungssysteme, wie z. B. Paysafecards oder Ukash.

29 https://www.bitkom.org/Presse/Presseinformation/Industrie-setzt-zunehmend-auf-Cyberversicherungen.html (zuletzt abgerufen am: 24.12.2021).

30 Trotz der 2017 veröffentlichten Musterbedingungen für eine „Cyberrisiko-Versicherung“, vgl. [18, S. 429 (430)].

31 *Gebert/Klapper*, in: [19, § 24 Rn. 24].

32 Allgemeine Versicherungsbedingungen für die Cyberrisiko-Versicherung (AVB Cyber), Musterbedingungen des GDV, Stand: April 2017.

33 https://www.finance-magazin.de/finanzabteilung/cybercrime/cyberversicherer-wir-lehnen-70-prozent-der-anfragen-ab-97457/ (zuletzt abgerufen am: 23.12.2021).
